# Prevalence of human schistosomiasis in various regions of Tanzania Mainland and Zanzibar: A systematic review and meta-analysis of studies conducted for the past ten years (2013–2023)

**DOI:** 10.1371/journal.pntd.0012462

**Published:** 2024-09-09

**Authors:** Nicolaus Omari Mbugi, Hudson Laizer, Musa Chacha, Ernest Mbega

**Affiliations:** 1 The Nelson Mandela African Institution of Science and Technology, School of Life Sciences and Bioengineering, Arusha, Tanzania; 2 Mbeya University of Science and Technology, College of Science and Technical Education, Mbeya, Tanzania; 3 Arusha Technical College, Arusha, Tanzania; Federal University of Agriculture Abeokuta, NIGERIA

## Abstract

Schistosomiasis is a significant public health problem in Tanzania, particularly for the people living in the marginalized settings. We have conducted a systematic review with meta-analysis on the prevalence of schistosomiasis to add knowledge towards the development of effective approaches to control the disease in Tanzania. Online databases namely, Pub Med, SCOPUS and AJOL, were systematically searched and a random effect model was used to calculate the pooled prevalence of the disease. Heterogeneity and the between studies variances were determined using Cochran (Q) and Higgins (I^2^) tests, respectively. A total of 55 articles met the inclusion criterion for this review and all have satisfactory quality scores. The pooled prevalence of the disease in Tanzania was 26.40%. Tanzania mainland had the highest schistosomiasis prevalence (28.89%) than Zanzibar (8.95%). Sub-group analyses based on the year of publication revealed the going up of the pooled prevalence, whereby for (2013–2018) and (2018–2023) the prevalence was 23.41% and 30.06%, respectively. The prevalence of the *Schistosoma mansoni* and *Schistosoma hematobium* were 37.91% and 8.86% respectively. Mara, Simuyu, and Mwanza were the most prevalent regions, with a pooled prevalence of 77.39%, 72.26%, and 51.19%, respectively. The pooled prevalence based on the diagnostic method was 64.11% for PCR and 56.46% for POC-CCA, which is relatively high compared to other tests. Cochrans and Higgins (I^2^) test has shown significant heterogeneity (p-value = 0.001 and I^2^ = 99.6). Factors including age, region, diagnostic method and sample size have shown significant contribution to the displayed heterogeneity. The pronounced and increasing prevalence of the disease suggests potential low coverage and possibly lack of involvement of some regions in the control of the disease. This, therefore, calls for an intensive implementation of control interventions in all endemic regions, preferably using an integrated approach that targets several stages of the disease lifecycle.

## Author summary

Schistosomiasis is a devastating tropical and sub-tropical disease that disproportionately infects those in resource-limited settings, which causes death, morbidity and socioeconomic impact. The disease is caused by blood parasites under the genus *Schistosoma*. Tanzania is one of the schistosomiasis burdened countries in the sub-Saharan region. Praziquantel mass drug administration has been a predominant schistosomiasis control strategy in the country. We have conducted a systematic review and meta-analysis using a prevalence dataset from published literature, aiming at assessing the country’s schistosomiasis burden (prevalence) following several years of disease control. We further conducted a subgroup analysis to assess the contributing factors to the observed disease prevalence. The obtained information provides insight into the impact of schistosomiasis control in Tanzania, and highlights the potential for positive change and improvement in the country’s fight against this disease.

## 1. Introduction

Schistosomiasis is one of the most pervasive tropical and sub tropical neglected diseases caused by blood helminths that belong to the genus *Schistosoma* [1,2]. This genus comprises several species that infect a vast range of hosts; however, only six are of clinical importance, namely *Schistosoma mansoni*, *Schistosoma japonicum*, *Schistosoma mekongi*, *Schistosoma guineansis*, *Schistosoma intercalatum* and *Schistosoma haematobium* [3]. The first five species cause intestinal schistosomiasis, whereas the latter causes urogenital schistosomiasis. Schistosomiasis is of public health importance due to its socioeconomic impact, ranking second after Malaria [4]. Recent data shows that over 237 million people are infected by schistosomiasis on a global scale, consequently accounting for 280,000 mortalities yearly [5,6,7]. Of all continents, Africa particularly the Sub-Saharan region, is leading in terms of disease burden, carrying 90% of all global cases [6]. In the region, Tanzania marks the second country in terms of disease burden, only surpassed by Nigeria [8,9]. Typically, *Schistosoma mansoni* and *Schistosoma hematobium* causing intestinal and urogenital schistosomiasis respectively, are endemic species in Tanzania, which cumulatively contribute to the pronounced prevalence [10]. Tanzania is the union of two countries, Zanzibar and Tanganyika, which was later referred to as Tanzania mainland. These two parts of the country have different backgrounds regarding schistosomiasis control. In Zanzibar, the history of schistosomiasis control dates back to the 1980s, when the disease prevalence, particularly urogenital schistosomiasis, was high [11]. The control of the disease was enforced through praziquantel mass drug administration in schools. This initiative was further enhanced by the Zanzibar Elimination of Schistosomiasis Transmission (ZEST) project between 2011 and 2017 [12, 13]. During the project, biannual praziquantel mass drug administration was implemented in both Pemba and Unguja islands to schools and the community at large. In addition, over the course of the project, other control interventions including bio-control of snails and behavioural changes were also employed [12]. Since the inception of the mentioned control interventions, there has been a considerable decrease in the disease burden from above 50% to 5% in 2020 in both islands. In view of this, current control initiatives focus on the complete elimination of the disease to make Zanzibar amongst the few regions in sub-Saharan Africa to achieve interruption of disease transmission [11].

Meanwhile, schistosomiasis control in Tanzania’s mainland dates back to 2000s, when the Schistosomiasis and Soil-transmitted Helminths Control Programme (NSSCP) was founded [14]. NSSCP is a joint partnership between the Ministry of Health, Community Development, Gender, Elderly and Children (MoHCDGEC) and the Ministry of Education and Vocational Training (MoEVT) established with the support of the Schistosomiasis Control Initiative (SCI). The programme has done much on disease surveillance and control through mass drug administration (MDA) using praziquantel. The praziquantel MDA mainly focused on school children, and from 2009 to 2018, more than 33.3 million drugs were delivered to the mentioned group [15]. The program covers all administrative regions that are endemic to schistosomiasis (about 17 regions), with a particular focus on the regions in a northern-western zone, which are highly endemic to the disease [15, 16]. In the regions that have prevalence > 50, the program was further extended to other high-risk groups [16]. However, the disease prevalence is still very high across endemic areas in the country, ranging from 12.7% to 87.6% [14]. The disease is more abundant in societies living along the shoreline of Lake Victoria, particularly in the Mwanza region, with vulnerable groups being pre-school and school-aged children as well as women[5]. The vulnerability of the aforementioned groups is attributed to their routine domestic activities that potentially expose them to infected water [7].

Both forms of schistosomiasis (urogenital and intestinal) are acquired through skin penetration of infective larvae (cercariae) into susceptible hosts[4]. This occurs when a susceptible host is exposed to infected water, and hence, infective larvae penetrate and migrate to their resident sites, where they grow into adult worms [3,4]. In the host body, adult worms reside in various destinations specific to the schistosome species, where they copulate and lay eggs [3,4]. The laid eggs are responsible for the pathophysiology of the disease, which is then clinically manifested in two forms; the acute and chronic manifestations [3,4]. Summarily, clinical manifestations of urogenital schistosomiasis include dysuria, nutritional deficiencies, haematuria, hydronephrosis, urinary bladder squamous cell carcinoma, and urinary bladder lesions [5]. For intestinal schistosomiasis, medical conditions such as splenomegaly, hepatomegaly, and progressive periportal fibrosis are manifested [5].

Considering the WHO guideline, the control of schistosomiasis in Tanzania and elsewhere predominantly relied on preventive chemotherapy by using Praziquantel drug. However, there is a need to assess the impact of this control intervention on the disease burden over the years of its implementation. Therefore, the present systematic review compiles epidemiological data on the prevalence of schistosomiasis in both Tanzania mainland and Zanzibar from 2013 to 2023. This will provide baseline information regarding the country’s response to Praziquantel mass drug administration as the mainstay for disease control, as well as provide the highlights for potential improvements in the fight against schistosomiasis.

## 2. Methodology

### 2.1 Data acquisition

Computer-assisted searches in online databases including Pub Med, SCOPUS and AJOL were done by using advanced search options to obtain relevant articles. MeSH term options from PubMed were usedf to obtain some terminologies used in the material search. Whereby, the search term used were Schistosomiasis OR Bilharzia* OR Schistosoma* OR Katayama* AND Epidemiology OR Prevalence AND Tanzania OR Zanzibar in combination. The obtained articles were primarily screened based on the relevance of their titles and abstracts to the reviewed topic by applying the prior defined inclusion and exclusion criterion. Duplicates were also removed using Mendeley. Again, full screenings were further performed to obtain the final set of publications used to compose the present review article.

### 2.2 Exclusion and inclusion criteria

Publications excluded from the use in the present review include publications not written in the English language, titles not relevant to the reviewed topic, reporting prevalence dataset from countries other than Tanzania, duplicating results of the research work from large project or research group, non-human studies, containing pooled datasets from two countries or more, contain datasets from a prospective longitudinal study following mass drug administration, as well as articles assessing genetic dynamics among both *S*. *hematobium* and *S*. *mansoni* populations. Whereas published articles containing prevalence data from the Tanzanian population and not returning visitors from Tanzania collected within the past 10 years (2013 to 2023), reporting baseline data from the intervention studies, prevalence data of schistosomiasis in various regions of Tanzania mainland and Zanzibar, assessing sensitivity of various diagnostic techniques with a clearly defined sample size and number of cases were included.

### 2.3 Articles selection and data extraction

Following combining results from the searched online databases, author NM and HL scanned all retrieved publications based on the titles and abstracts for their eligibility to be fully reviewed. Eligible articles were full text reviewed by two independent authors (NM and HL) for their inclusion illegibility in data extraction. Articles from both authors that are matching were selected for data extraction. For the case of any mismatch other two authors (MC and EM) were consulted. Prior to data extraction, all duplicates were removed. Afterwards, data were independently abstracted by two authors (NM and HL) using a pre-designed form in Microsoft Excel. The form was pre-tested by all authors (NM, HL, MC, EM) prior to its use in data extraction. On the occasion that there was a difference in data extracted by the two authors from similar articles, other two authors were invited (MC and EM) to extract the data independently. Similar data extracted by at least three of the four authors was taken. Extracted data includes the author’s name, year of publication, study regions, diagnostic method used, sample size (study population), number of cases, target groups, study design used as well as the age range (Table 1).

### 2.4 Assessment of study quality and risk of bias

Quality and risk of bias for the selected publications were evaluated on the basis of criteria stipulated in the Joanna Briggs Institute critical appraisal checklist for use in reviews of prevalence studies[17]. Briefly, each criterion was accredited 1 grade if a criterion was met and 0 if a criterion was not met. Nine (9) was the maximum score given when all criteria were met, whereas 0 was the minimum score given to an article when none of the criteria were met. Articles scored cumulative grades ranging from 0–4 were regarded are of low quality, 5–7 moderate quality and 8–9 high quality. After that, articles demonstrating moderate to high quality were included in the present systematic review and meta-analysis. Authors (NM and HL) independently evaluated the quality of included publications.

### 2.5 Meta-analysis

Meta-analysis was done in line with the previously published protocol by employing a random effects model [17]. Through this, point estimates of the weighted prevalence of the datasets from the included studies were computed at 95% confidence intervals (CIs) and presented by forest plot. Heterogeneity, as well as variation between studies was assessed using Cochran’s Q (chi-square) and (Higgins) I^2^ tests, respectively. I^2^ values above 50% were considered to indicate substantial heterogeneity. For Cochran’s Q (chi-square) test, the heterogeneity was considered significant at p-value = 0.1. The univariate subgroup analysis to assess the contributing factors to the observed heterogeneity was computed with regard to the year of publication, region, diagnostic method, sample size, age of the participants and schistosoma species. The mentioned factors were grouped in the following order; year of publication into 2 groups; (2013–2018) and (2018–2023), regions into 17 groups; Ruvuma, Lindi, Mwanza, Dar es salaam, Morogoro, Mara, Simiyu, Shinyanga, Geita, Zanzibar, Kilimanjaro, Kagera, Dodoma and combination of regions such as (Kagera, Mara and Mwanza), (Mwanza and Shinyanga), (Mwanza and Kagera) and (Kagera, Mara, Shinyanga and Mwanza). Sample size was categorized into 3 categories; <100 (small), 100–499 (moderate) and >500 (large), age of the participant into 3 categories; <18, (18 and above) and all ages, schistostosoma species into 2 categories; *S*.*mansoni* and *S*. *haematobium*, and diagnostic method into 12 categories; Kato katz, PCR, POC-CCA, Mini FLOTAC, Sedimentation, Microscopy, Direct wet preparation, Urine filtration, Urine centrifugation, CAA, Direct smear and microhematuria. The factors demonstrating significant heterogeneity was subjected to multivariate subgroup analysis and the amount contributed by each factor or combination of significant factors (R^2^) was determined.

Additionally, publication bias was examined visually using a funnel plot and the degree of asymmetry was further confirmed by using Egger’s regression test. Whereby, it was assumed that the symmetrical distribution of the study effect sizes across the plot indicates the absence of the study bias. In contrast, the asymmetrical distribution reflects the presence of the study bias. All statistical analyses were done in R software version 4.3.0 using the meta prop function under the meta and metaphor package.

## 3 Results

### 3.1 Literature search results

#### 3.1.1 Characteristics of the reviewed publications

**Fig 1:**
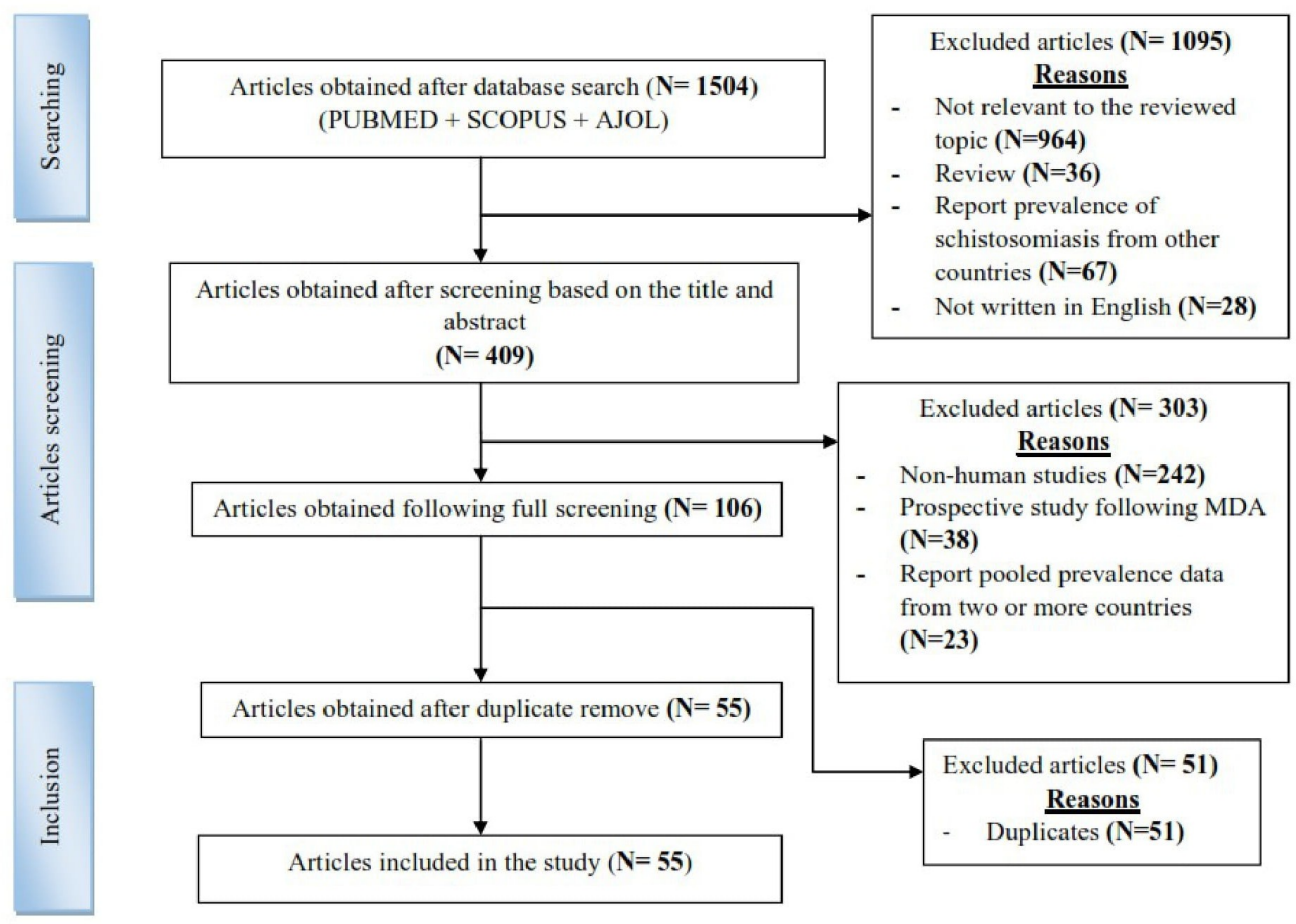
Conceptual framework of the literature search and screening process.

A systematic literature search produced 1504 publications ranging from 2013 to 2023, from which only 55 met the inclusion criteria by reporting on the prevalence datasets from disparate regions of Tanzania and hence included in the present review work (Fig 1). Of the included publications, 3 reported prevalence datasets from women, 4 from preschool-aged children, 22 from school-aged children, 3 from both pre and school-aged children, 2 from both children and adults, 2 from children, 1 from HIV-infected children, 1 from psychiatric patients, 1 from adults with TB, 2 from adults with HIV, 1 from street children and orphans, 10 from adults, 3 from the general population, 1 from secondary school students and 1 from the university students (Table 1). On the other hand, the study design from the selected 55 publications was 44 cross-sectional studies, 1 case-control, 2 cohorts, 5 prospective longitudinal, 1 randomized controlled trial, and 2 study designs were not specified (Table 1).

Among the selected publications, 30 assessed the prevalence of *S*. *mansoni*, 15 of *S*. *hematobium*, and 11 of both *S*. *hematobium* and *S*. *mansoni*. The regions from Tanzania were reported as follows; 1 publications report prevalence dataset from Ruvuma, 3 from Lindi, 3 from Mara, 23 from Mwanza, 5 from Dar es Salaam, 2 from Simiyu, 5 from Zanzibar, 2 from Shinyanga, 1 from Geita, 1 from Kagera, 4 from Morogoro, 2 from Dodoma and 1 from Kilimanjaro. Meanwhile, 2 publications report combined datasets from Kagera, Mara, Shinyanga and Mwanza, 1 from Mwanza and Shinyanga and 1 from Mwanza and Kagera (Table 1).

An array of diagnostic methods were used to screen both *S*. *mansoni* and *S*. *hematobium* whereby, Kato katz was the mostly used diagnostics method as reported by 30 studies, followed by urine filtration (n = 19), POC-CCA (n = 19), PCR (n = 5), microscopy (n = 4), urine concentration (n = 3), direct smear (n = 2), formal ether concentration (n = 3), CAA (n = 3), microhematuria (n = 1) and Mini FLOTAC (n = 2) (Table 1). The enrolled studies recruited a total of 122,674 individuals from Zanzibar as well as 12 regions of Tanzania mainland. The age of examined individuals ranges from 0 to 95 years.

**Table 1:** General characteristics of the reviewed articles.

Author	Year of publication	Study area	Study design	Targeted species	Targeted group	Age (Years)	Diagnostic methods	Sample size	Cases
Angelo et al.[18]	2018	Shinyanga	Longitudinal	*S*. *hematobium*	School-aged children	12 to 14	Urine filtration	282	98
Bakuza et al.[19]	2018	Lindi	Cross-sectional	*S*. *hematobium*	Children	9 to 12	Urine filtration	190	44
Barda et al.[20]	2013	Mwanza	Unspecified	*S*. *mansoni*	Children	4 to 19	Direct smear	201	8
Barda et al. [20]	2013	Mwanza	Unspecified	*S*. *mansoni*	Children	4 to 19	Kato Katz	201	66
Barda et al. [20]	2013	Mwanza	Unspecified	*S*. *mansoni*	Children	4 to 19	Mini FLOTAC	201	98
Barda et al.[21]	2014	Mwanza	Cross-sectional	*S*. *mansoni*	School children and adults	Above 5	Mini FLOTAC	251	58
Barda et al. [21]	2014	Mwanza	Cross-sectional	*S*. *mansoni*	School children and adults	Above 5	Formol ether concentration	251	32
Barda et al. [21]	2014	Mwanza	Cross-sectional	*S*. *mansoni*	School children and adults	Above 5	Direct smear	251	9
Barda et al. [21]	2014	Mwanza	Cross-sectional	*S*. *mansoni*	School children and adults	Above 5	Urine concentration	151	72
Bukindu et al.[22]	2016	Mwanza	Cross-sectional	*S*. *mansoni*	School-aged children	8 to 18	Kato Katz	625	229
Casacuberta et al. [23]	2016	Mwanza	Cross-sectional	*S*. *mansoni*	School-aged children	9 to 12	Kato Katz	404	303
Casacuberta et al. [23]	2016	Mwanza	Cross-sectional	*S*. *mansoni*	School-aged children	9 to 12	POC-CCA	404	172
Fanz et al. [24]	2023	Mwanza	Cross-sectional	*S*. *mansoni*	Orphans	6 to 18	POC-CCA	144	91
Fanz et al. [24]	2023	Mwanza	Cross-sectional	*S*. *mansoni*	Street children	6 to 18	POC-CCA	122	103
Fanz et al.[24]	2023	Mwanza	Cross-sectional	*S*. *mansoni*	Orphans	6 to 18	Kato Katz	144	28
Fanz et al. [24]	2023	Mwanza	Cross-sectional	*S*. *mansoni*	Street children	6 to 18	Kato Katz	112	86
Fulgence et al. [25]	2023	Dar es Salaam	Cross-sectional	*S*. *mansoni*	University students	19 to 33	Kato Katz	272	22
Fuss et al.[26]	2018	Mwanza	Cross-sectional	*S*. *mansoni*	School-aged children	7 to 16	Kato Katz	297	253
Fuss et al. [26]	2018	Mwanza	Cross-sectional	*S*. *mansoni*	School-aged children	7 to 16	POC-CCA	297	282
Fuss et al. [26]	2018	Mwanza	Cross-sectional	*S*. *mansoni*	School-aged children	7 to 16	Real-time PCR	297	276
Fuss et al.[27]	2020	Mwanza	Cross-sectional	*S*. *mansoni*	Adults	18 to 70	Kato Katz	36	12
Fuss et al. [27]	2020	Mwanza	Cross-sectional	*S*. *mansoni*	Adults	18 to 70	POC-CCA	36	23
Fuss et al. [27]	2020	Mwanza	Cross-sectional	*S*. *mansoni*	Adults	18 to 70	Serum real-time PCR	36	27
Fuss et al. [27]	2020	Mwanza	Longitudinal	*S*. *mansoni*	Adults	18 to 70	Urine real-time PCR	36	11
Fuss et al. [28]	2021	Mwanza	Cross-sectional	*S*. *mansoni*	Adults	17 to 70	POC-CCA	100	80
Fuss et al. [28]	2021	Mwanza	Cross-sectional	*S*. *mansoni*	Adults	17 to 70	Serum real-time PCR	95	84
Fuss et al. [28]	2021	Mwanza	Cross-sectional	*S*. *mansoni*	Adults	17 to 70	DBS real-time PCR	100	41
Fuss et al.[28]	2021	Mwanza	Cross-sectional	*S*. *mansoni*	Adults	17 to 70	Kato Katz	98	43
Kaatano et al. [29]	2015	Mwanza	Cross-sectional	*S*. *mansoni*	Adults	12 to 85	Kato Katz	388	121
Kapiga et al. [30]	2021	Mwanza and Kagera	Cross-sectional	*Schistosoma spp*.	Adult	Above 18	CAA	1112	924
Kayange et al.[31]	2020	Mwanza	Cross-sectional	*S*. *mansoni*	School-aged children	6 to 13	POC-CCA	507	253
Kayange et al. [31]	2020	Mwanza	Cross-sectional	*S*. *hematobium*	School-aged children	6 to 13	Urine filtration	507	8
Keller et al.[32]	2020	Zanzibar	Cross-sectional	*S*. *hematobium*	Children and adults	9 to 55	qPCR(Dra1 DNA)	792	212
Keller et al. [32]	2020	Zanzibar	Cross-sectional	*S*. *hematobium*	Children and adults	9 to 55	Urine filtration	792	105
Keller et al. [32]	2020	Zanzibar	Cross-sectional	*S*. *hematobium*	Children and adults	9 to 55	Microhaematuria	792	109
Kinug’hi et al.[33]	2014	Mwanza	Cross-sectional	*S*. *mansoni*	Pre-school and school-aged children	3 to 13	Kato Katz	1,546	613
Kinug’hi et al. [33]	2014	Mwanza	Cross-sectional	*S*. *hematobium*	Pre-school and school-aged children	3 to 13	Nucleopore filtration method	1,546	305
Kinunghi et al.[34]	2017	Mara	Cross-sectional	*S*. *mansoni*	School-aged children	6 to 15	Kato Katz	928	794
Kisiringyo et al. [35]	2020	Morogoro	Cross sectional	*S*. *hematobium*	School children	5 to 16	Formal-ether sedimentation	374	186
Kisiringyo et al. [35]	2020	Morogoro	Cross sectional	*S*. *mansoni*	School children	5 to 16	Formal-ether sedimentation	374	1
Knopp et al. [36]	2015	Zanzibar	Unspecified	*S*. *hematobium*	School-aged children	8 to12	Urine filtration	1740	58
Knopp et al. [37]	2018	Zanzibar	Cross-sectional	*S*. *hematobium*	Adults	20 to 55	Urine filtration	18,155	490
Knopp et al. [37]	2018	Zanzibar	Cross-sectional	*S*. *hematobium*	Children	9 to 12	Urine filtration	39,207	2,117
Masikini et al. [38]	2019	Mwanza	Case-control	*S*. *mansoni*	HIV infected adults	Above 18	Microscopy	170	19
Masikini et al. [38]	2019	Mwanza	Case-control	*S*. *mansoni*	HIV infected adults	Above 18	CAA	188	82
Mazigo et al.[39]	2019	Mwanza	Cross-sectional	*S*. *mansoni*	HIV infected children	1 to 16	Kato Katz	103	11
Mazigo et al. [39]	2019	Mwanza	Cross-sectional	*S*. *mansoni*	HIV infected children	1 to 16	POC-CCA	134	45
Mazigo et al.[40]	2021	Mwanza	A prospective longitudinal	*S*. *mansoni*	School-aged children	7 to 17	Kato Katz	399	226
Mazigo et al. [40]	2021	Mwanza	A prospective longitudinal	*S*. *mansoni*	School-aged children	7 to 17	POC-CCA	399	398
Mazigo et al.[41]	2017	Mara	Cross-sectional	*S*. *mansoni*	Adults	18 to 89	Kato Katz	412	232
Mazigo et al.[42]	2014	Mwanza	Cross-sectional	*S*. *mansoni*	Adults	21 to 55	Kato Katz	1,785	854
Mazigo et al.[5]	2021	Ruvuma	Cross-sectional	*S*. *hematobium*	Pre and school-aged children	1 to 13	Urine filtration	1,560	13
Mazigo et al. [5]	2021	Ruvuma	Cross-sectional	*S*. *mansoni*	Pre and school-aged children	1 to 13	Kato Katz	1,560	236
Mazigo et al. [5]	2021	Ruvuma	Cross-sectional	*S*. *mansoni*	Pre and school-aged children	1 to 13	POC-CCA	574	125
Mazigo et al. [43]	2018	Mwanza	Prospective longitudinal study	*S*. *mansoni*	General population	15 to 55	Kato Katz	419	242
Mazigo et al. [43]	2018	Mwanza	Prospective longitudinal study	*S*. *mansoni*	General population	15 to 55	POC-CCA	419	365
Mazigo et al.[44]	2018	Mwanza	Cross-sectional	*S*. *mansoni*	HIV infected adults	15 to 55	Kato Katz	979	463
Mazigo et al. [44]	2018	Mwanza	Cross-sectional	*S*. *mansoni*	HIV infected adults	15 to 55	POC-CCA	979	592
Mhimbira et al.[45]	2017	Dar es Salaam	Cohort	*S*. *mansoni*	Children and adults with TB	above 18	POC-CCA	597	55
Mhimbira et al. [45]	2017	Dar es Salaam	Cohort	*S*. *hematobium*	Children and adults with TB	above 18	Urine filtration	597	19
Mnkugwe et al.[46]	2020	Simiyu	Cross-sectional	*S*. *hematobium*	School-aged children	5 to 19	Kato Katz	830	752
Mohamed et al. [47]	2018	Mwanza	Cross-sectional	*S*. *mansoni*	School-aged children	9 to 11	Kato Katz	327	103
Mueller et al.[48]	2019	Mwanza	Cross-sectional	*S*. *mansoni*	General population	1 to 95	Kato Katz	930	641
Mueller et al. [48]	2019	Mwanza	Cross-sectional	*S*. *mansoni*	General population	1 to 95	POC-CCA	930	879
Mugono et al.[49]	2014	Mwanza	Cross-sectional	*S*. *mansoni*	School-aged children	4 to 15	Kato Katz	773	494
Munisi et al.[50]	2016	Mara	Cross-sectional	*S*. *mansoni*	School-aged children	6 to 16	Kato Katz	513	431
Mushi et al. [8]	2022	Lindi	Cross-sectional	*S*. *hematobium*	Preschool aged Children	under 5	Urine filtration	385	65
Mushi et al. [51]	2022	Lindi	Cross-sectional	*S*. *hematobium*	School-aged children	6 to 17	Urine filtration	649	342
Ndokeji et al.[52]	2016	Mwanza	Cross-sectional	*S*. *mansoni*	Pre-school and School-aged children	4 to 14	Kato Katz	454	363
Ng’weng’weta et al. [53]	2017	Dar es Salaam	Cross-sectional	*S*. *hematobium*	Pre-school children	6 to 7	Urine centrifugation	424	8
Ngasala et al. [54]	2019	Dodoma	Cross-sectional	*S*. *hematobium*	School-aged children	5 to 16	Microscopy	353	24
Ngasala et al. [54]	2019	Zanzibar	Cross-sectional	*S*. *hematobium*	School-aged children	7 to 14	Microscopy	150	58
Ngassa et al. [55]	2023	Kilimanjaro	Cross-sectional	*S*. *hematobium*	Women	15 to 45	Sedimentation	216	5
Nkya [56]	2023	Morogoro	Cross sectional	*S*. *hematobium*	School children	6 to 16	Microscopy	884	287
Nyundo et al.[57]	2017	Dodoma	Cross-sectional	*S*. *mansoni*	Psychiatric patients	12 to 69	Direct wet preparation and formol-ether concentration	233	12
Ogweno et al. [58]	2023	Simiyu	Cross-sectional	*S*. *mansoni*	School-aged children	Unspecified	Kato Katz	363	150
Palmeirim et al.[59]	2021	Morogoro	Cross-sectional	*S*. *mansoni*	School-aged children	6 to12	POC-CCA	427	53
Pham et al. [60]	2023	Mwanza	Cross-sectional	*Schistosoma spp*.	Adults	Above 18	CAA	1,923	873
Rite et al. [61]	2020	Geita	Cross-sectional	*S*. *hematobium*	Women	15 to 49	Urine filtration	426	19
Rosinger et al.[62]	2018	Mwanza and Shinyanga	Cross-sectional	*S*. *hematobium*	Non-pregnant women	18 to 50	Urine filtration	209	12
Rosinger et al. [62]	2018	Mwanza and Shinyanga	Cross-sectional	*S*. *mansoni*	Non-pregnant women	18 to 50	Kato Katz	205	11
Ruganuza et al.[63]	2015	Mwanza	Cross-sectional	*S*. *mansoni*	Pre-school children	1 to 6	Kato Katz	400	178
Ruganuza et al. [63]	2015	Mwanza	Cross-sectional	*S*. *mansoni*	Pre-school children	1 to 6	POC-CCA	400	320
Said et al.[64]	2017	Dar es Salaam	Prospective longitudinal	*Schistosoma spp*.	Pre-school aged children	Under 5	POC-CCA	308	47
Said et al. [64]	2017	Dar es Salaam	Prospective longitudinal	*S*. *hematobium*	Pre-school aged children	Under 5	Urine filtration	308	3
Samweli et al. [65]	2023	Shinyanga	Cross-sectional	*S*. *mansoni*	Secondary school students	11 to 20	Kato Katz	620	12
Shabani et al. [66]	2022	Kagera	Cross sectional	*S*. *mansoni*	Adults	18 to 55	Formal-ether sedimentation	328	36
Sikalengo et al.[67]	2018	Dar es Salaam	Cohort study	*S*. *mansoni*	Adult TB patients	Above 18	Stool microscopy	460	8
Sikalengo et al. [67]	2018	Dar es Salaam	Cohort study	*S*. *mansoni*	Adult TB patients	Above 18	POC-CCA	460	19
Sikalengo et al. [67]	2018	Dar es Salaam	Cohort study	*S*. *hematobium*	Adult TB patients	Above 18	Urine filtration	460	16
Sikalengo et al. [67]	2018	Morogoro	Cohort study	*S*. *mansoni*	Adult TB patients	Above 18	Stool microscopy	208	7
Sikalengo et al. [67]	2018	Morogoro	Cohort study	*S*. *mansoni*	Adult TB patients	Above 18	POC-CCA	208	34
Sikalengo et al. [67]	2018	Morogoro	Cohort study	*S*. *hematobium*	Adult TB patients	Above 18	Urine filtration	208	3
Siza et al.[68]	2015	Kagera, Mara, Shinyanga and Mwanza	Cross-sectional	*S*. *mansoni*	School-aged children	7 to 16	Kato Katz	5,952	898
Siza et al. [68]	2015	Kagera, Mara, Sinyanga and Mwanza	Cross-sectional	*S*. *hematobium*	School-aged children	7 to 16	Urine filtration	5,826	519
Siza et al.[69]	2015	Kagera, Mara and Mwanza	Cross-sectional	*S*. *mansoni*	Adults	Not specified	Kato Katz	1,606	199
Siza et al. [69]	2015	Kagera, Mara and Mwanza	Cross-sectional	*S*. *mansoni*	Adults	Not specified	Urine filtration	1,400	25
Wang et al.[70]	2019	Zanzibar	A randomized controlled trial	*S*. *hematobium*	General population	Unspecified	Urine filtration	6,000	175
Yangaza et al. [71]	2020	Dar es salaam	Cross sectional	*S*. *hematobium*	School children	7 to 15	Urine filtration	250	3

### 3.2 Quality and risk of bias assessment

Examination of the study quality and risk of bias revealed the absence of low-quality studies; 23 publications had moderate quality hence scored (5–7), whereas 32 studies had high quality with score range of (8–9). The average score was 7.57, which indicates the overall moderate quality of the included publications. All studies used standard procedures of sample collection (stool, urine and blood) as well as valid diagnostic tests for the detection of both urogenital and intestinal schistosomiasis.

### 3.3 Pooled prevalence and sub-group analysis

**Fig 2:**
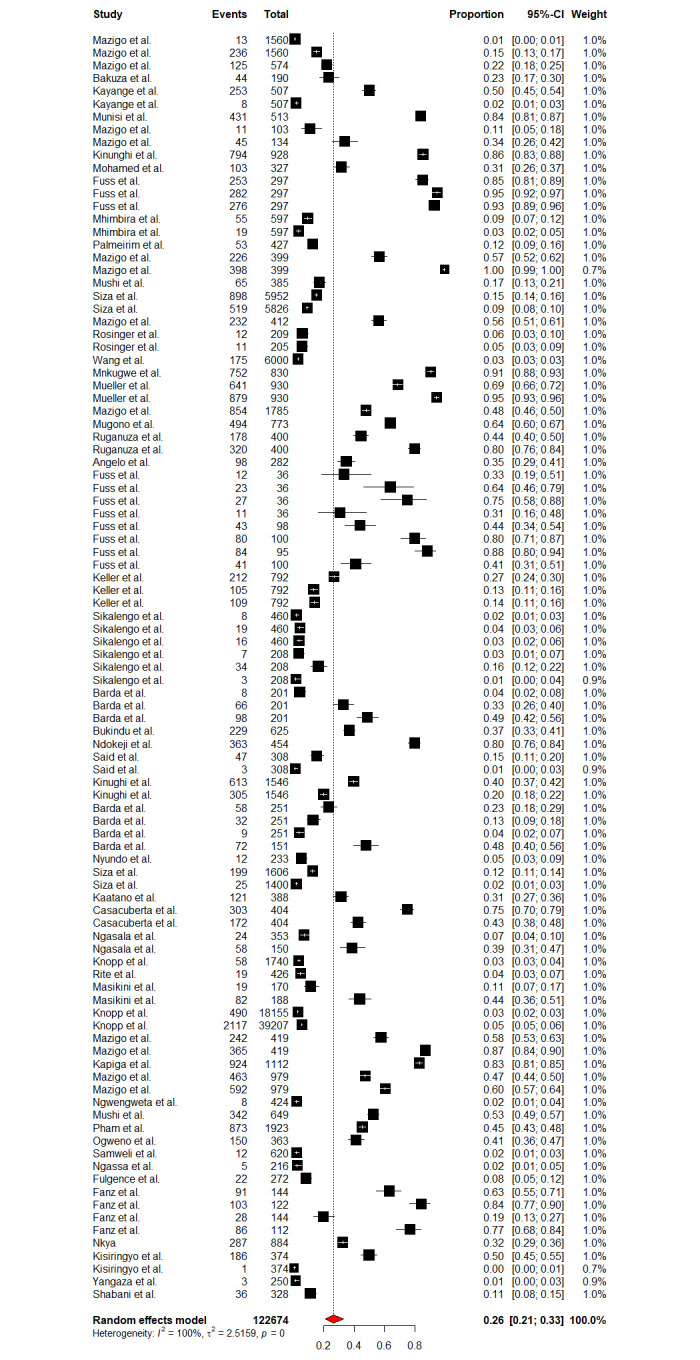
Forest plot showing the pooled prevalence of schistosomiasis in Tanzania.

Fifty-five (55) studies have reported on schistosomiasis prevalence datasets from various regions of Tanzania mainland and Zanzibar, which were used to estimate the country pooled prevalence. The pooled prevalence of schistosomiasis (both urogenital and intestinal) in Tanzania was 26.40% [95% CI: 20.73–32.98, *I*^*2*^ *=* 98.5%] (Fig 2). Cochran’s Q test portrayed substantial heterogeneity at p-value = 0.001, meanwhile, the between study variances determined using Higgins (I^2^) was also very high (99.6%).

**Fig 3:**
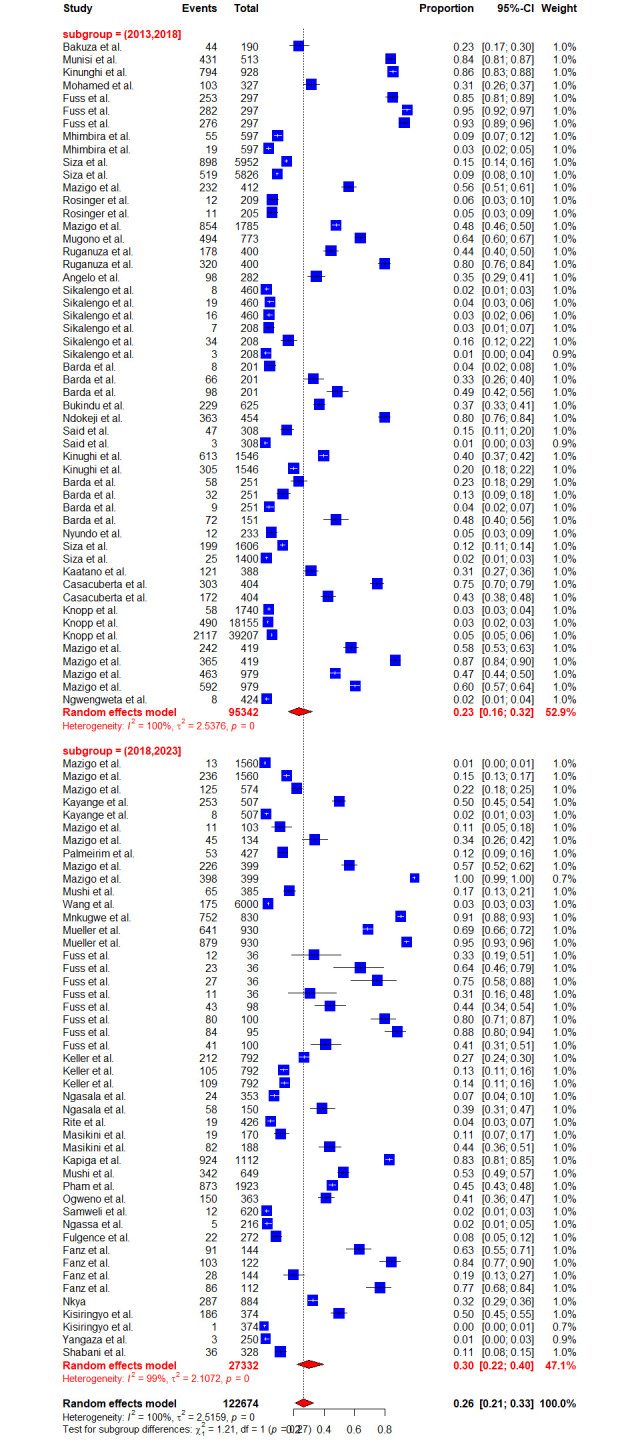
Forest plot showing the pooled prevalence of schistosomiasis in years of publication subgroups.

**Fig 4:**
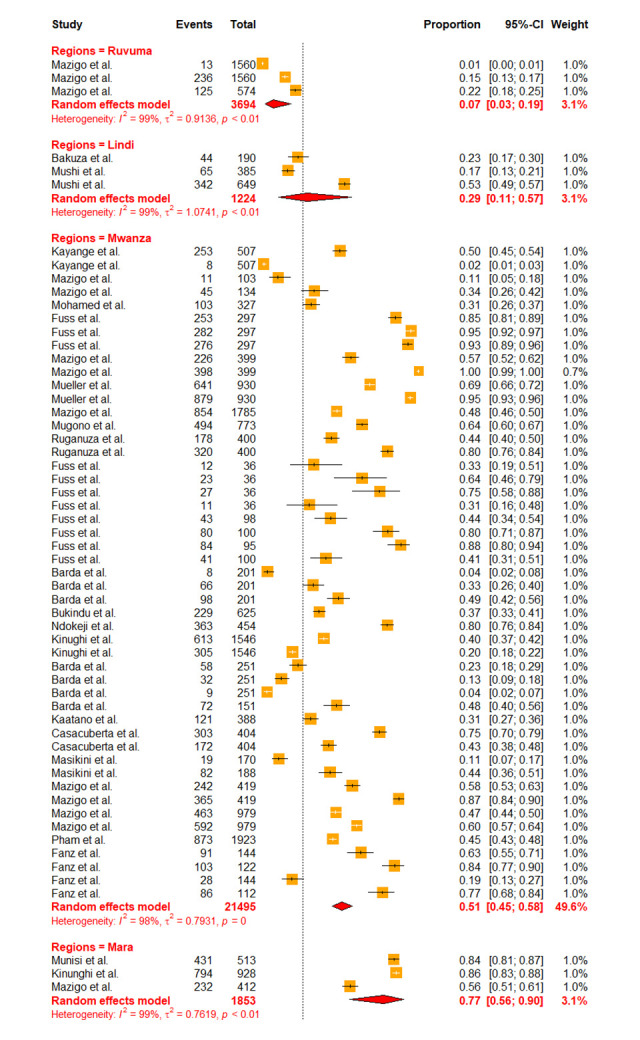
Forest plot showing the pooled prevalence of schistosomiasis in region sub-group.

**Fig 5:**
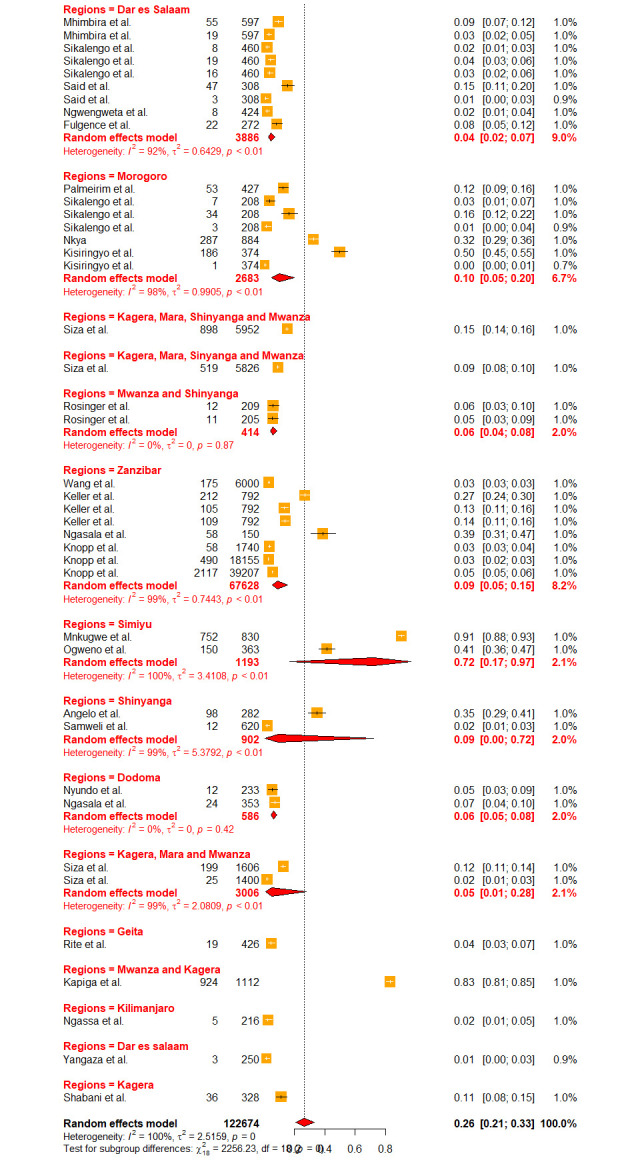
Forest plot showing the pooled prevalence of schistosomiasis in region sub-group.

**Fig 6:**
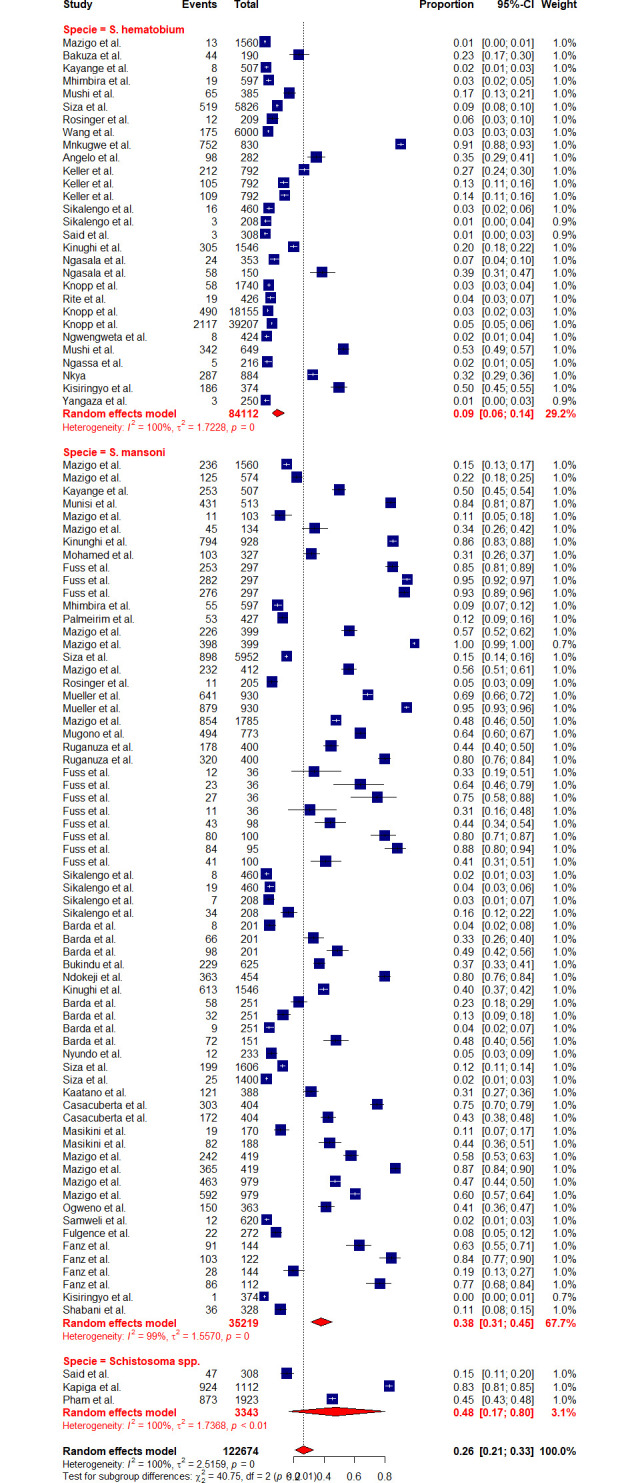
Forest plot showing the pooled prevalence of schistosomiasis based on specie sub-group.

**Fig 7:**
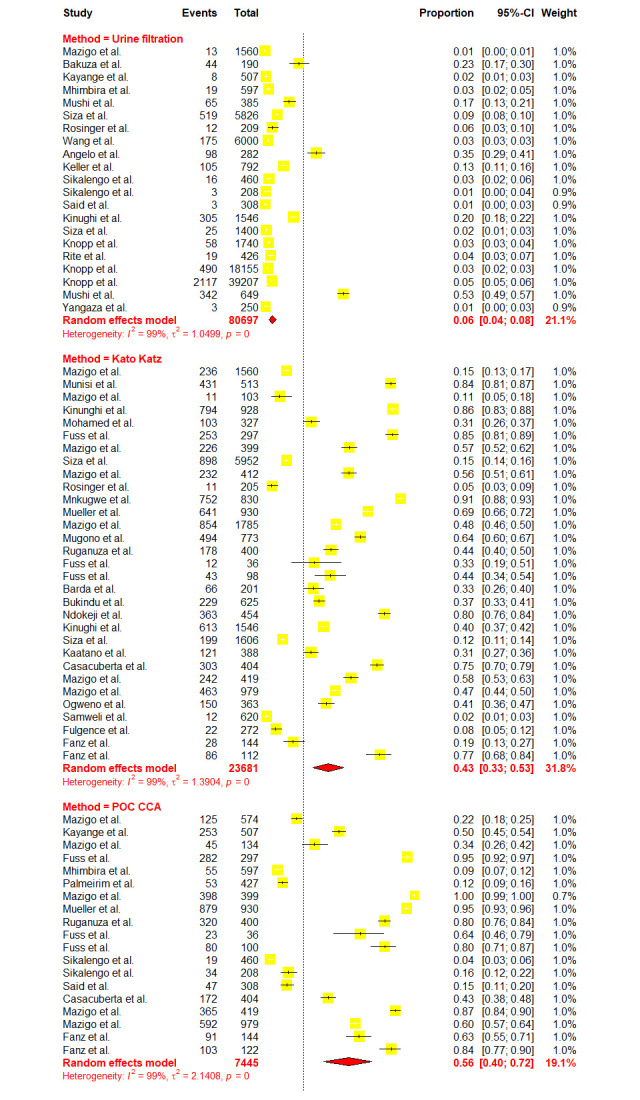
Forest plot showing the pooled prevalence of schistosomiasis based on the diagnostic method.

**Fig 8:**
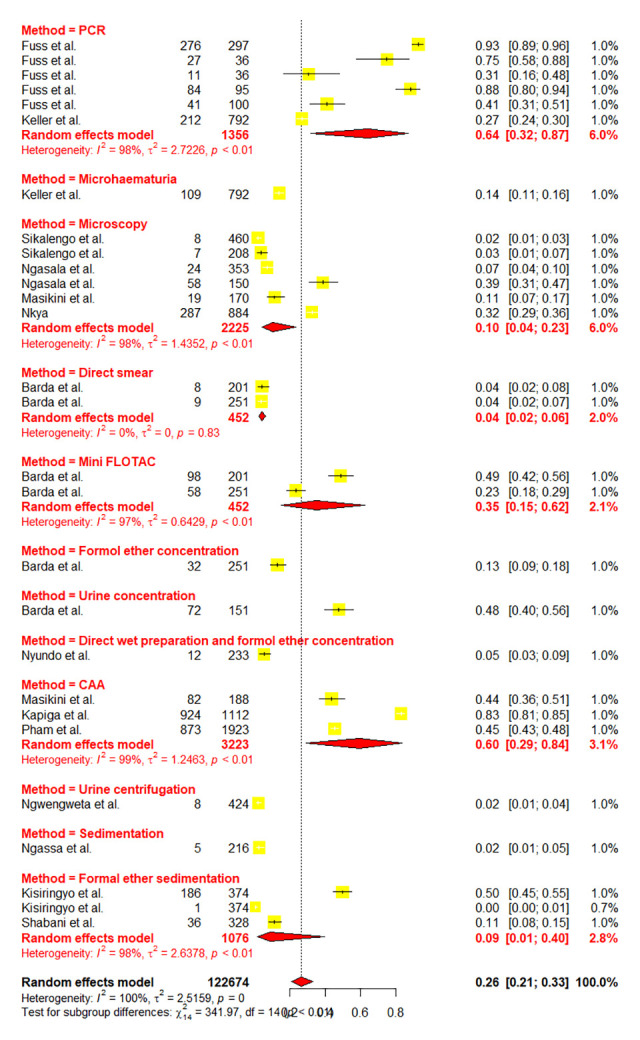
Forest plot showing the pooled prevalence of schistosomiasis based on the diagnostic method.

**Fig 9:**
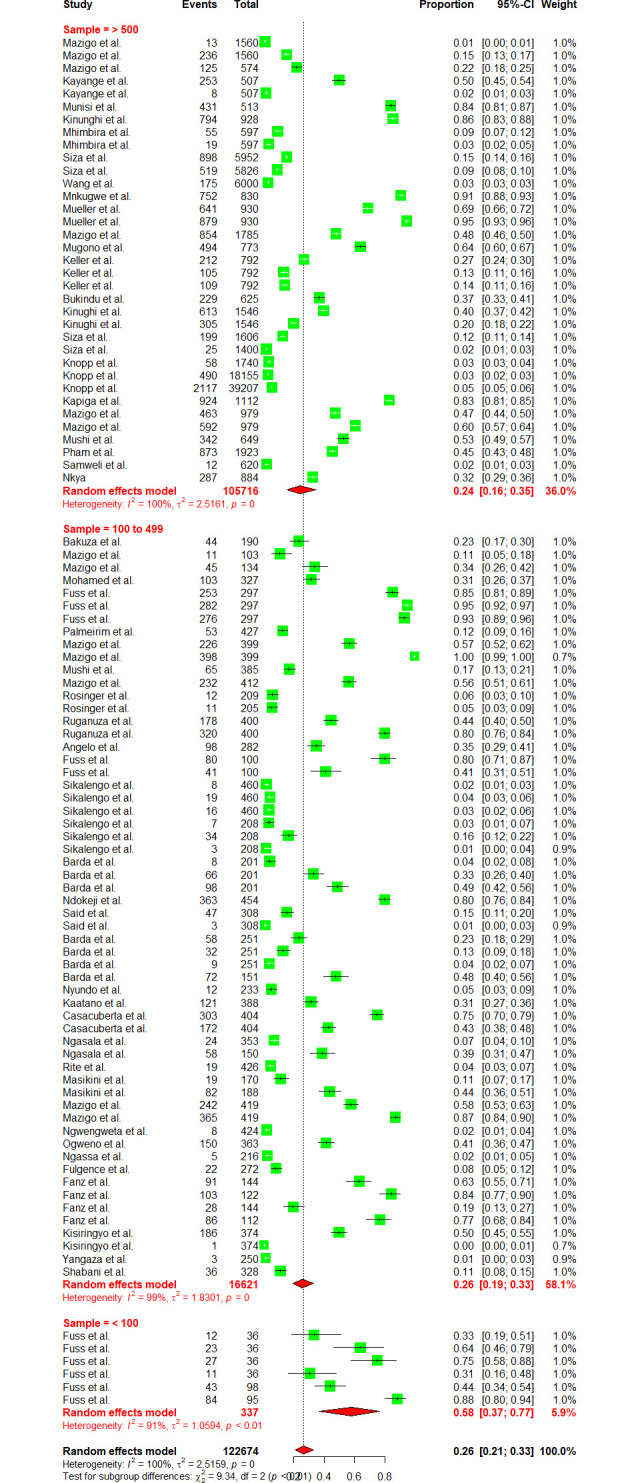
Forest plot of the sub group analysis based on the sample size.

**Fig 10:**
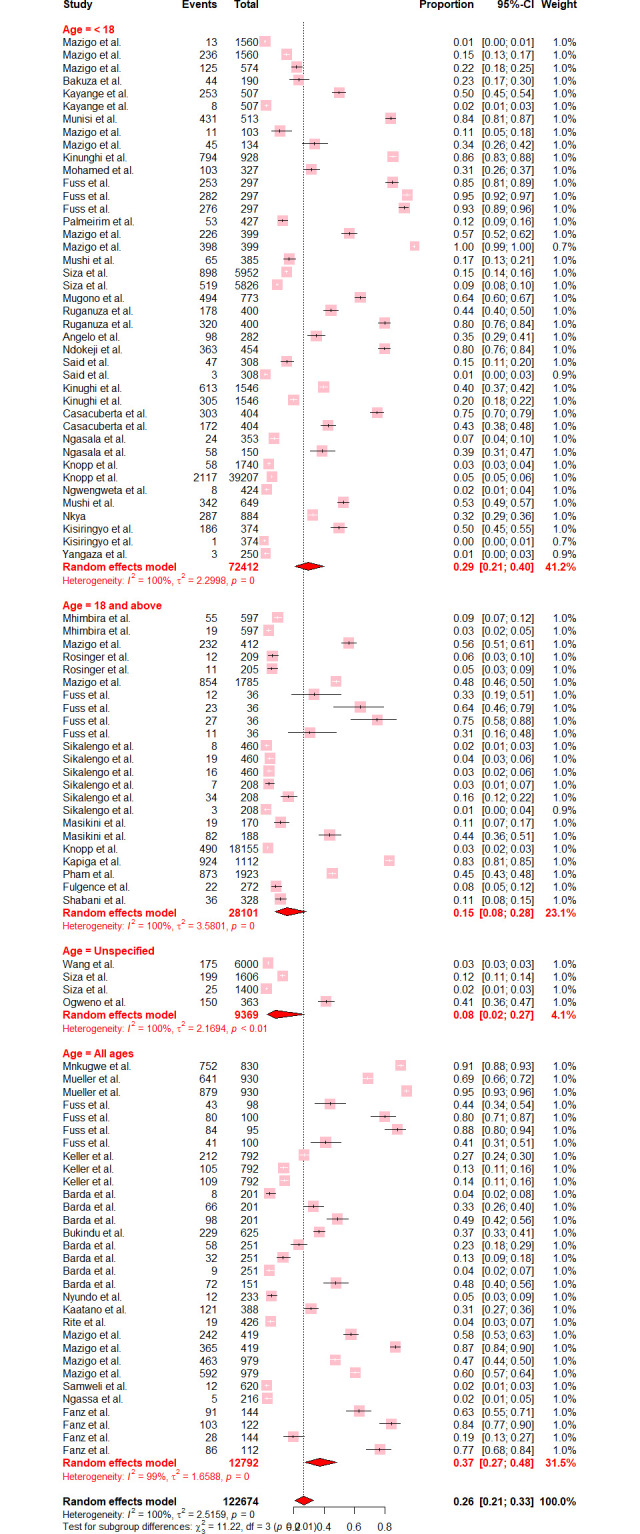
Plot of the sub group analysis based on the participants ages.

Due to high heterogeneity, sub-group analyses were conducted to assess the effects of the contributing factors, which include Schistosoma species, year of publication, diagnostic methods, sample size, participant’s age as well as regions. Sub-group analysis based on the years of publication did not reveal significant heterogeneity at p-value = 0.2716, whereas other assessed factors such as diagnostic methods (p-value = < 0.0001), sample size (p-value = 0.0094), participant’s ages (p-value = < 0.0106), regions (p-value = 0.001) and schistosomes specie (p-value = < 0.0001) have shown significant contribution to the observed high heterogeneity. Furthermore, based on the years of publication, the sub-group analysis revealed the pooled prevalence of 23.41% [95% CI: 16.49–32.11] and 30.06% [95% CI: 21.97–39.61] at the following time intervals (2013–2018) and (2018–2023) respectively (Fig 3). Sub-group analysis based on the regions revealed the highest prevalence of schistosomiasis in Mara, Simiyu, and Mwanza with a prevalence of 77.39% [95% CI: 55.86–90.25, *I*^*2*^ *=* 98.5%], 72.26% [95% CI: 16.70–97.13, *I*^*2*^ *=* 99.6%], and 51.19% [95% CI: 44.79–57.56, *I*^*2*^ *=* 98.5%] respectively (Figs 4 and 5). Sub-group analysis of the prevalence dataset based on the Schistosoma species divulged the pooled prevalence of 8.86% [95% CI: 5.64–13.65, *I*^*2*^ = 99.5%] and 37.91% [95% CI: 31.05–45.29, *I*^*2*^ = 99.2%] for *S*. *hematobium* and *S*. *mansoni* respectively (Fig 6). Furthermore, sub-group analysis based on the diagnostic method showed the highest prevalence of PCR and POC-CCA with a pooled prevalence of 64.11 [95% CI: 31.89–87.21, *I*^*2*^ = 98.4%], 56.45 [95% CI 37.68–73.53, *I*^*2*^ = 99.1%] respectively (Fig 7 and 8). Estimated pooled prevalence based on the sample size sub group were 23.83 [95% CI: 15.59–34.64, *I*^*2*^ = 99.8%], 25.54 [95% CI: 19.38–32.87, *I*^*2*^ = 98.5%] and 0.5795 [95% CI: 0.3670–0.7660, *I*^*2*^ = 91.4%] for the sample size >500, 100–499 and <100 respectively (Fig 9). For the sub group analysis on the basis of the participant ages, the estimated prevalence of schistosomiasis were 29.47 [95% CI: 20.68–40.10, *I*^*2*^ = 99.7%], 15.19 [95% CI: 7.58–28.12, *I*^*2*^ = 99.6%] and 37.21 [95% CI: 27.24–48.40, *I*^*2*^ = 99.0%] for the following age groups; < 18, 18 and above and all ages respectively (Fig 10). The results of multivariate sub group analysis revealed that, factors including age (R^2^ = 100%), sample size (R^2^ = 100%), diagnostic method (R^2^ = 100%) and Region (R^2^ = 100%) contribute strongly to the overall heterogeneity (variability observed in the effect sizes) (p-value = 0.0001).

**Fig 11:**
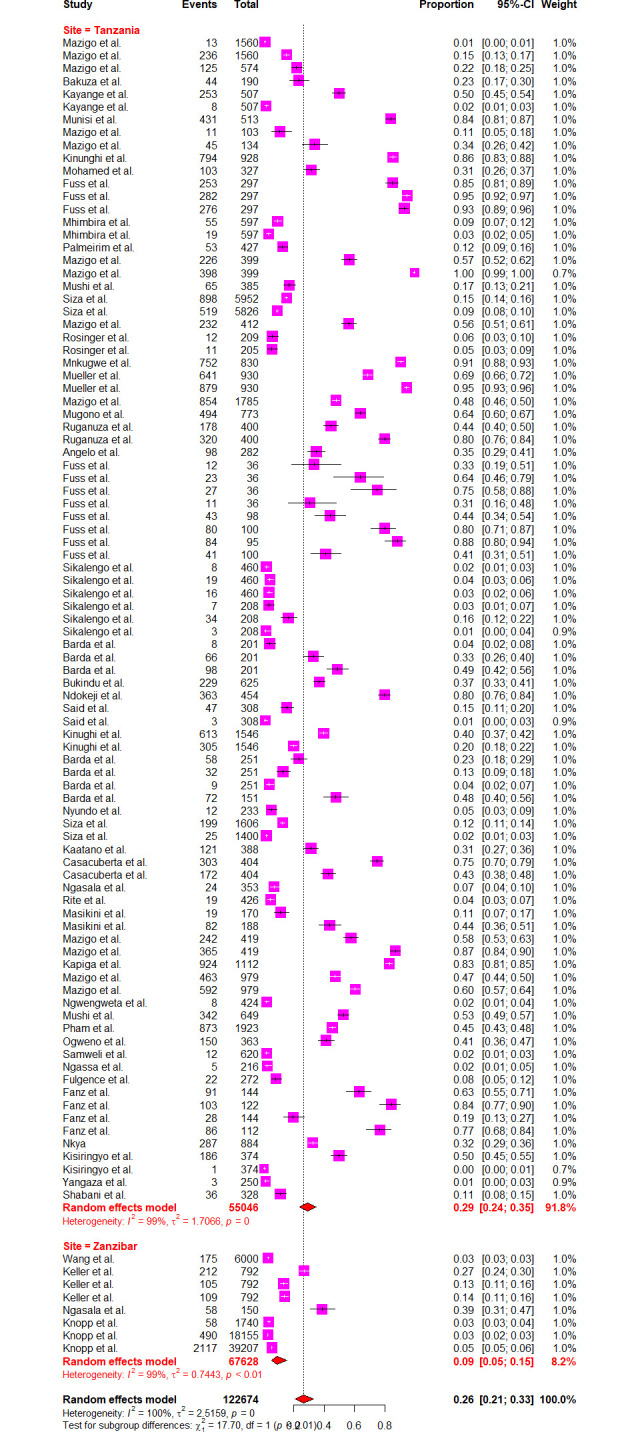
Forest plot showing the pooled prevalence of schistosomiasis in Tanzania mainland and Zanzibar.

Since Zanzibar and Tanzania mainland have disparate histories regarding schistosomiasis control, additional sub group analysis were conducted to determine the estimated pooled prevalence of the mentioned areas. The estimated prevalence of schistosomiasis in Tanzania mainland was 28.89% [95% CI: 23.61–0.3482, *I*^*2*^ = 99.3%]; meanwhile, for Zanzibar was 8.95% [95% CI 5.11–15.22, *I*^*2*^ = 99.4%] (Fig 11).

### 3.4 Publication bias

**Fig 12:**
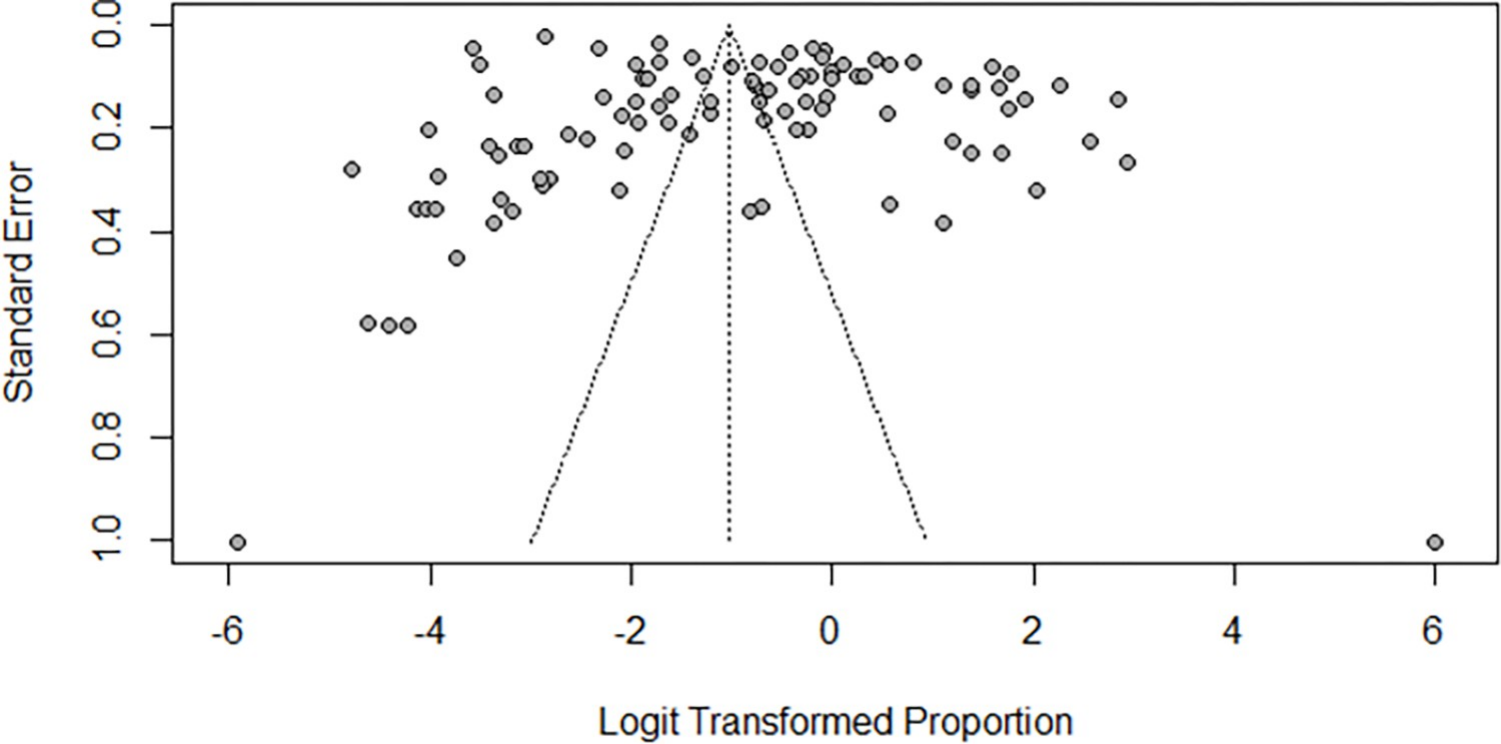
Funnel plot showing the symmetrical distribution of studies along the plot.

Publication bias was assessed using a funnel plot of the effect sizes against standard error; as such, there was a symmetrical distribution of studies effect sizes along the plot, indicating the absence of the publication bias (Fig 12). However, egger’s regression analysis for funnel plot asymmetry outcome fails to confirm the absence of publication bias (p-value = 0.0099).

## 4. Discussion

Schistosomiasis (both urogenital and intestinal) is an endemic disease in the Sub-Saharan region and the most devastating parasitic disease behind Malaria. Tanzania, being located in this region, is also highly affected by schistosomiasis. The present review assessed the prevalence of schistosomiasis in various regions of Tanzania mainland and Zanzibar and reported their pooled estimate values (prevalence). To the best of our knowledge, this is the first review reporting the prevalence of schistosomiasis in Tanzania for the past ten years (2013–2023).

The meta-analysis of the dataset from included studies revealed a high pooled prevalence of Schistosomiasis in Tanzania (26.40%). Notably, the prevalence was higher in Tanzania mainland than in Zanzibar. The lower prevalence in Zanzibar can be attributed to the successful implementation of the praziquantel mass drug administration strategy, which was carried out biannually for over 6 years [12,13]. Other strategies, such as behavioural changes and bio-control of the disease intermediate host, also contributed to the reduction of the disease burden [12,13]. However, the high prevalence in the mainland suggests that some regions may not have responded effectively to the control interventions or were not sufficiently involved in the treatment strategies, leading to the overall rise in prevalence.

The degree of heterogeneity and between study variances were high and attributed to factors including sample size, regions, diagnostic method, schistosoma species, and participant age. The prevalence of intestinal Schistosomiasis was high compared to urogenital schistosomiasis. The high prevalence of intestinal schistosomiasis can be attributed to the tendency of people to defecate near water bodies due to the presence of long vegetation and the availability of water for cleaning themselves, as was previously reported by Zacharia and coworkers (2020). This, therefore, fosters the spread of the disease through exposure of susceptible individuals to the infected water. The observed high prevalence of intestinal schistosomiasis is supported by the study done by Zacharia and coworkers (2020), which reported the re-infection rate and the prevalence of intestinal schistosomiasis at the global scale being higher relative to that of urogenital schistosomiasis [17]. The five-year interval analysis of schistosomiasis prevalence revealed a significant increase. As explained above, this surge may indicate inconsistency and lack of sustainability in the implementation of disease control interventions, as some regions might not have effectively responded to or have ceased implementing the treatment strategies, leading to a rise in overall prevalence. The north-western zone, which comprises regions including Mara, Simiyu, and Mwanza, has a pronounced pooled prevalence of the disease. The observed high prevalence could be affiliated with the presence of Lake Victoria, which is a potential source of transmission, especially when people conduct their socioeconomic activities such as fishing, agriculture, laundry, bathing, and mining, among others, thereby increasing the risk of subsequent re-infection [10].

On the other hand, proper and timely detection of schistosomiasis is an imperative step toward the efficient elimination of the disease. Failure to correctly diagnose the disease will, therefore, compromise the intensive work conducted to control the disease. Sub-group analysis based on the diagnostic methods divulged the high prevalence of schistosomiasis detected by PCR and POC-CCA. This indicates that the aforementioned methods are highly sensitive and, indeed, effective for the detection of both urogenital and intestinal schistosomiasis. As such, they should be widely adopted in the detection of schistosomiasis, as recommended by Bisetegn and coworkers, 2021 [72]. For sub group analysis on the basis of the participant age, it was revealed that participants of all ages were the most infected group. The high prevalence of this age group is because it encompasses all vulnerable groups, including school aged children, women of reproductive age, fishermen, etc., whose probability of exposure to infected water is relatively high. This observation agrees with another study that reported a high re-infection rate of schistosomiasis in individuals of all ages compared to other age groups [17]. Studies with small sample sizes had a high prevalence compared to those with moderate and large sample sizes. The reason behind the high prevalence of schistosomiasis in studies with small sample sizes could be the easy and intensive follow up compared to the ones with large sample sizes. Visual examination of the funnel plot portrayed the absence of the study bias as the study effect sizes were symmetrically distributed across the plot. However, eggers regression test outcomes failed to confirm the absence of the plot asymmetry; hence, it indicates the potential missing out of some studies, particularly the ones with small effect sizes.

## 5. Conclusion and recommendation

Despite extensive efforts put in place by the government of Tanzania in trying to eliminate schistosomiasis as a complement to the sustainable development goal number 3 of attaining health and wellbeing for all by 2030, the disease prevalence is still growing, as stipulated in this review. The regions surrounding Lake Victoria had the higher pooled prevalence and were considered the core hub for subsequent re-infection of schistosomiasis. The review also highlights that *S*. *mansoni* is the most prevalent species in Tanzania relative to *S*. *hematobium*. In view of that, an intensive deployment of praziquantel mass drug administration in combination with other control strategies in the endemic regions, particularly in the mainland part, is of paramount importance. The present study also proves that PCR and POC-CCA are the most sensitive methods for the detection of both urogenital as well as intestinal schistosomiasis as compared to the commonly used microscopy and direct smear methods. Given this, the aforementioned methods should be adopted during disease management interventions as the methods of choice for the identification of disease cases.
