# Supplementary material for: Prevalence of human schistosomiasis in various regions of Tanzania Mainland and Zanzibar: A systematic review and meta-analysis of studies conducted for the past ten years (2013–2023)
Source: PLoS Negl Trop Dis. 2024 Sep 9;18(9):e0012462. doi: 10.1371/journal.pntd.0012462 (PMC11412511; doi:10.1371/journal.pntd.0012462)
Supplement: Attachment — Submitted filename: Comments.docx [file pntd.0012462.s001.docx]

Thank you for the opportunity to review this important manuscript. The systematic review and meta-analysis will provide crucial evidence of the prevalence of schistosomiasis in Tanzania, which as the country has changed its target from morbidity control to elimination of the disease as public health problem. To improve the manuscript I have the following comments.

1. Abstract and main text: I noticed the use of improper terminologies such as “management” instead of “intervention” and “eradication” instead of “control” or “elimination as public health problem” or “interruption of transmission”. This is because for example, it is inappropriate to use the term "eradication" because the majority of endemic countries have not reached that stage and no schistosomiasis eradication strategies have been established in most of them. "Control" or "eliminate as a public health problem" are appropriate terminologies, however, in few countries “interruption of transmission” may apply.
2. Abstract: *S. mansoni* and *S. haematobium* should be written in full in the first mention.
3. Main text: Are *Schistosoma guineansis* and *Schistosoma intercalatum* the same species? If not, why they have been grouped as one?
4. Main text: The two sides of Tanzania (mainland and Zanzibar) have different story on interventions approaches, previous and current status and achievements regarding schistosomiasis control. Authors need to provide these information in the introduction section and explain how this study will be useful to the current approaches used by the two sides of the country.
5. Main text: The authors explained that “the present systematic review compiles epidemiological data on the prevalence of schistosomiasis in Tanzania from 2013 to 2023”. However, in their inclusion criteria they considered dates of publication rather than the dates of data collection. For example, data presented in the studies by Mwakitalu et al [13], Kinughi et al [11], Khan et al [23] and Manz et al [12] were collected between May 2006 and October 2012.
6. Main text: Authors have included only two databases (PubMed and SCOPUS) but there other databases such Web of Science, HINARI, AJOL and Google Scholar. Don’t authors think that they may have lost other important articles by excluding these databases?
7. Main text: Authors did not provide initials of author(s) who did literature search, study selection and data extraction, and how they did it i.e. independently or as a team.
8. Main text: Figure 1, authors need to provide reasons for exclusion of the studies in each stage.
9. Main text: Assessment of study quality and risk of bias is important aspect of review and meta-analysis studies. It seems that authors did not perform it as there is no information provided in the manuscript. I suggest authors to check the quality of the included studies and presence of any risk of bias.
10. Provide reference for the statement “Meta-analysis was done in line with the previously published protocol by employing a random effects model.”
11. Main text: Sample size for each study, study population and study setting are among of important factors that have been considered as potential source of heterogeneity in meta-analysis studies. I suggest authors to include them in the subgroup analysis.
12. Main text: Authors did not give the cut off for the Higgins I^2^ to indicate the presence or absence of substantial heterogeneity. Please provide the cut off value.
13. I suggest authors to use the Cochran’s Q (chi-square) test which is also good indicator of the level of heterogeneity during meta-analysis.
14. Main text: To my understanding, if more than one factors are considered for subgroup analysis, it should be performed in two stages. The first stage is the univeraite subgroup analysis where authors needs to assess the contribution of each factor independently. At this stage the authors will determine the amount of heterogeneity contributed by each factor (not presented), the level of significance (not presented) and the pooled prevalence of each category (presented). Then, factors that showed significant amount of heterogeneity should be subjected to multivariate subgroup analysis to determine the amount of heterogeneity contributed by combination of those factors and the level of significance. I suggest authors to follow these stages.
15. Main text: In the methodology section, authors explained that they will use Egger’s regression test to confirm the funnel plot asymmetry results, but no results for this test presented in the results section. Please provide the results.
16. Main text: Since Zanzibar has its own schistosomiasis control program with difference control approaches, historical perspective, current status and achievement. I think it will be perfect to consider it separately. Otherwise, authors may have another subgroup consisting of two categories; mainland Tanzania and Zanzibar.
17. Main text: “To the best of our knowledge, this is the first review reporting the prevalence of schistosomiasis in Tanzania for the past ten years”. See comment number 5.
18. Main text: In the discussion section replace “…..several rounds of disease control interventions” with “…..several rounds of praziquantel mass drug administration”. Also, provide reference for this statement.
19. Revise all references to adhere to the journal format.
