# Supplementary material for: Prevalence of human schistosomiasis in various regions of Tanzania Mainland and Zanzibar: A systematic review and meta-analysis of studies conducted for the past ten years (2013–2023)
Source: PLoS Negl Trop Dis. 2024 Sep 9;18(9):e0012462. doi: 10.1371/journal.pntd.0012462 (PMC11412511; doi:10.1371/journal.pntd.0012462)
Supplement: Attachment — Submitted filename: Author response to reviewers comments.docx [file pntd.0012462.s002.docx]

**Author response to reviewer’s comments**

|  | **Reviewer comment** | **Response** |
| --- | --- | --- |
| 1. | Abstract and main text: I noticed the use of improper terminologies such as “management” instead of “intervention” and “eradication” instead of “control” or “elimination as public health problem” or “interruption of transmission”. This is because for example, it is inappropriate to use the term "eradication" because the majority of endemic countries have not reached that stage and no schistosomiasis eradication strategies have been established in most of them. "Control" or "eliminate as a public health problem" are appropriate terminologies, however, in few countries “interruption of transmission” may apply. | All inappropriate words were replaced as recommended by reviewers in both abstract and the main text |
| 2. | Abstract: *S. mansoni* and *S. haematobium* should be written in full in the first mention. | All names were written correctly |
| 3. | Main text: Are *Schistosoma guineansis* and *Schistosoma intercalatum* the same species? If not, why they have been grouped as one? | *Schistosoma guineansis* and *Schistosoma intercalatum* were separated as independent species |
| 4. | Main text: The two sides of Tanzania (mainland and Zanzibar) have different story on interventions approaches, previous and current status and achievements regarding schistosomiasis control. Authors need to provide these information in the introduction section and explain how this study will be useful to the current approaches used by the two sides of the country. | A brief history of the schistosomiasis control in Tanzania mainland and Zanzibar were provided in the introduction section. |
| 5. | Main text: The authors explained that “the present systematic review compiles epidemiological data on the prevalence of schistosomiasis in Tanzania from 2013 to 2023”. However, in their inclusion criteria they considered dates of publication rather than the dates of data collection. For example, data presented in the studies by Mwakitalu et al [13], Kinughi et al [11], Khan et al [23] and Manz et al [12] were collected between May 2006 and October 2012. | All included studies which their data were collected before 2013 were eliminated |
| 6. | Main text: Authors have included only two databases (PubMed and SCOPUS) but there other databases such Web of Science, HINARI, AJOL and Google Scholar. Don’t authors think that they may have lost other important articles by excluding these databases? | We added AJOL in our articles searching |
| 7. | Main text: Authors did not provide initials of author(s) who did literature search, study selection and data extraction, and how they did it i.e. independently or as a team. | Author’s initials and their contribution to this work were added. |
| 8. | Main text: Figure 1, authors need to provide reasons for exclusion of the studies in each stage. | Reasons added |
| 9. | Main text: Assessment of study quality and risk of bias is important aspect of review and meta-analysis studies. It seems that authors did not perform it as there is no information provided in the manuscript. I suggest authors to check the quality of the included studies and presence of any risk of bias. | All included studies were assessed for their quality and risk of bias by using criterion described in Joanna Briggs Institute critical appraisal checklist for use in reviews of prevalence studies |
| 10. | Provide reference for the statement “Meta-analysis was done in line with the previously published protocol by employing a random effects model | Reference added |
| 11. | Main text: Sample size for each study, study population and study setting are among of important factors that have been considered as potential source of heterogeneity in meta-analysis studies. I suggest authors to include them in the subgroup analysis. | Sample size and study population were included in the sub-group analysis |
| 12. | Main text: Authors did not give the cut off for the Higgins I^2^ to indicate the presence or absence of substantial heterogeneity. Please provide the cut off value | Cut off for the Higgins I^2^ was indicated |
| 13. | I suggest authors to use the Cochran’s Q (chi-square) test which is also good indicator of the level of heterogeneity during meta-analysis. | Cochran’s Q (chi-square) were used to asses heterogeneity between studies |
| 14. | Main text: To my understanding, if more than one factors are considered for subgroup analysis, it should be performed in two stages. The first stage is the univeraite subgroup analysis where authors needs to assess the contribution of each factor independently. At this stage the authors will determine the amount of heterogeneity contributed by each factor (not presented), the level of significance (not presented) and the pooled prevalence of each category (presented). Then, factors that showed significant amount of heterogeneity should be subjected to multivariate subgroup analysis to determine the amount of heterogeneity contributed by combination of those factors and the level of significance. I suggest authors to follow these stages. | The procedure was followed. However, we still stand to be corrected |
| 15. | Main text: In the methodology section, authors explained that they will use Egger’s regression test to confirm the funnel plot asymmetry results, but no results for this test presented in the results section. Please provide the results. | Result for eggers regression test was added |
| 16. | Main text: Since Zanzibar has its own schistosomiasis control program with difference control approaches, historical perspective, current status and achievement. I think it will be perfect to consider it separately. Otherwise, authors may have another subgroup consisting of two categories; mainland Tanzania and Zanzibar | Sub group analysis for Zanzibar and Tanzania mainland were conducted |
| 17. | Main text: “To the best of our knowledge, this is the first review reporting the prevalence of schistosomiasis in Tanzania for the past ten years”. See comment number 5. | Done |
| 18. | Main text: In the discussion section replace “…..several rounds of disease control interventions” with “…..several rounds of praziquantel mass drug administration”. Also, provide reference for this statement. | Replacement were done as per reviewers recommendation |
| 19. | Revise all references to adhere to the journal format. | All references were revised |
| 20. | Other comments;   - Removing of unnecessary words such as “on the other hand”, “ whereby” and “concurrently” - Removing the word “endemic” from the first sentence in the introduction section - Line numbering - Using primary source during citation - Changing the picture format to TIF as well as checking the quality using PACE - Addition of the “author summary” - Proper numbering of the table (s) | - All unnecessary words were removed as recommended by reviewers - The word endemic were replaced by the words “tropical and sub-tropical” disease - Line numbers were added - Primary source were used for citation as recommended by reviewers - The format of the uploaded pictures were converted to TIF files and diagnosed by PACE to see if they meet PLOS requirement - Author summary (Synopsis) were added - Tables were properly numbered |
