# Supplementary material for: Prevalence of human schistosomiasis in various regions of Tanzania Mainland and Zanzibar: A systematic review and meta-analysis of studies conducted for the past ten years (2013–2023)
Source: PLoS Negl Trop Dis. 2024 Sep 9;18(9):e0012462. doi: 10.1371/journal.pntd.0012462 (PMC11412511; doi:10.1371/journal.pntd.0012462)
Supplement: Attachment — Submitted filename: Comments.docx [file pntd.0012462.s003.docx]

Thank you again for the opportunity to review this important manuscript. I see the current manuscript has great improvement compare to the previous one and the authors have worked on most of the comments, however I have the following few new comments and noted some previous comments that were not correctly addressed by authors:

1. Line 112: to achieve interruption of the disease what??? Transmission, pathogenesis….????
2. Line 114: change “….were founded” to “…..was founded”
3. Line 104 – 120: Could authors provide epidemiological and geographical coverage of Praziquantel MDA to either sides of the country?
4. Line 136: Replace the word “management” with “control”.
5. Line 161 “reporting potential schistosomiasis transmission sites” and line 162-3 “distribution, and abundances of schistosomiasis intermediate host (freshwater snails)”, these should be summarized as “non-human studies”.
6. Line 165: Add the word “published” just before the word “articles”
7. Line 166: Replace the word “published” with “collected”.
8. To make good flow of information I suggest the subsection “assessment of quality and risk of bias” should come after the subsection “article selection and data extraction”.
9. Line 202 – 204: Authors should provide more details on the subgroup analysis. How was the factors categorized? How the analysis, both univeraite and multivariate analysis were done? What criteria was used for the factors to be considered for the multivariate analysis?
10. Figure 1, authors had provided the reasons for excluding articles, but they didn’t indicate the specific number of articles excluded for each reason. I suggest them to add it if possible.
11. Line 226 – 228: the statement “The two common forms of schistosomiasis, i.e. urogenital and intestinal schistosomiasis are caused by *S. hematobium* and *S. mansoni* respectively, and both are prevalent in East Africa including Tanzania” should be moved to either introduction or discussion section.
12. Line 242: Table 1 column 2 presents “year”. This column title is not specific to be more specific I suggested the authors to revise it. If authors have presented year of publication they should explain it.
13. Line 242: Table 1, I suggested the authors to presented both year of publication and year of data collection in two different columns.
14. Line 260: Be specific year of what?
15. Line 259 – 288: I think the sub-group analysis results presented are for univeraite. If so, I expected to see results of multivariate sub-group analysis for the factors with significant results in univeraite analysis i.e. diagnostic methods, age, and region. Also, the amount of heterogeneity (R^2^) contributed by each factors (univeraite analysis) and combination of factors (multivariate analysis) were not presented. Please read careful your reference review protocol on how to analyse and present results of subgroup analysis (pages 4, 5, 7 and 9).
16. Line 305: No text have been presented for subsection 4.3 funnel plot for bias analysis.
17. Line 341-343:”The high prevalence observed indicates that the rate of reinfection is still very high despite the implementation of several rounds of praziquantel mass drug administration” This statement should be supported by praziquantel MDA coverage information (epidemiological and geographical coverage).
18. Reduce repetition of results in the discussion section.
