# Supplementary material for: Prevalence of human schistosomiasis in various regions of Tanzania Mainland and Zanzibar: A systematic review and meta-analysis of studies conducted for the past ten years (2013–2023)
Source: PLoS Negl Trop Dis. 2024 Sep 9;18(9):e0012462. doi: 10.1371/journal.pntd.0012462 (PMC11412511; doi:10.1371/journal.pntd.0012462)
Supplement: Attachment — Submitted filename: Author response to reviewers comments.docx [file pntd.0012462.s004.docx]

**Author response to reviewer’s comments**

|  | **Reviewer comment** | **Response** |
| --- | --- | --- |
| 1. | Line 112: to achieve interruption of the disease what??? Transmission, pathogenesis….???? | Word “transmission” was added to the sentence |
| 2. | Line 114: change “….were founded” to “…..was founded” | Word “were” was replaced by “was” |
| 3. | Line 104 – 120: Could authors provide epidemiological and geographical coverage of Praziquantel MDA to either sides of the country? | The information regarding epidemiological and geographical coverage were added in the introduction section |
| 4. | Line 136: Replace the word “management” with “control”. | The word “management” was replaced “control” |
| 5. | Line 161 “reporting potential schistosomiasis transmission sites” and line 162-3 “distribution, and abundances of schistosomiasis intermediate host (freshwater snails)”, these should be summarized as “non-human studies”. | The two mentioned reasons were summarized as recommended |
| 6. | Line 165: Add the word “published” just before the word “articles” | The word “published” was added as recommended |
| 7. | Line 166: Replace the word “published” with “collected”. | The word “published” was replaced by the word “collected” |
| 8. | To make good flow of information I suggest the subsection “assessment of quality and risk of bias” should come after the subsection “article selection and data extraction”. | The sub- sections were rearranged as recommended |
| 9. | Line 202 – 204: Authors should provide more details on the subgroup analysis. How was the factors categorized? How the analysis, both univeraite and multivariate analysis were done? What criteria was used for the factors to be considered for the multivariate analysis? | The information was added as recommended by reviewer |
| 10. | Figure 1, authors had provided the reasons for excluding articles, but they didn’t indicate the specific number of articles excluded for each reason. I suggest them to add it if possible | Number of excluded articles were added |
| 11. | Line 226 – 228: the statement “The two common forms of schistosomiasis, i.e. urogenital and intestinal schistosomiasis are caused by *S. hematobium* and *S. mansoni* respectively, and both are prevalent in East Africa including Tanzania” should be moved to either introduction or discussion section. | The statement were moved to introduction section as suggested by reviewers |
| 12. | Line 242: Table 1 column 2 presents “year”. This column title is not specific to be more specific I suggested the authors to revise it. If authors have presented year of publication they should explain it. | We specified “year of publication” |
| 13. | Line 242: Table 1, I suggested the authors to presented both year of publication and year of data collection in two different columns. | It is a good suggestion but we thought that if we add this column, the table will be congested with a lot of information. However, in sub group meta analysis we used “year of publication” which is presented in the table |
| 14. | Line 260: Be specific year of what? | “Year of publication” was added to the text to make it specific |
| 15. | Line 259 – 288: I think the sub-group analysis results presented are for univeraite. If so, I expected to see results of multivariate sub-group analysis for the factors with significant results in univeraite analysis i.e. diagnostic methods, age, and region. Also, the amount of heterogeneity (R^2^) contributed by each factors (univeraite analysis) and combination of factors (multivariate analysis) were not presented. Please read careful your reference review protocol on how to analyse and present results of subgroup analysis (pages 4, 5, 7 and 9). | Multivariate analysis of the contributing factors was done and the amount contributed by each factor was presented as (R^2^) |
| 16. | Line 305: No text have been presented for subsection 4.3 funnel plot for bias analysis. | Subsection 4.3 was removed and figure 10 was referred in the subsection 3.4 |
| 17. | Line 341-343:”The high prevalence observed indicates that the rate of reinfection is still very high despite the implementation of several rounds of praziquantel mass drug administration” This statement should be supported by praziquantel MDA coverage information (epidemiological and geographical coverage). | The information was added in the introduction section |
| 18. | Reduce repetition of results in the discussion section. | Repetition were reduced in the discussion section as recommended by reviewers |
| 19. | Other comments   1. Correct the word “Specie” 2. Change the word time-dependent increase and re-infection rate 3. Remove word generally 4. Remove the words metaprop function 5. Remove the sentence “this age group” 6. Summarize the abstract to be 300 words 7. Remove statement “there is emerging treatment resistance in S. mansoni” 8. p value of 0 9. corrections on some reference | 1. Word “specie” was corrected to “species” 2. Words “ time-dependent increase” and “re-infection rate” were change as recommended by reviewers 3. The word generally was removed as recommended 4. The words metaprop function were removed 5. The sentence “this age group” was removed as suggested 6. The abstract was summarized to 300 words 7. The statement “there is emerging treatment resistance in S. mansoni” was removed 8. P value of zero were corrected to 0.001 9. Corrections on the reference were done |
