# Supplementary material for: Prevalence of human schistosomiasis in various regions of Tanzania Mainland and Zanzibar: A systematic review and meta-analysis of studies conducted for the past ten years (2013–2023)
Source: PLoS Negl Trop Dis. 2024 Sep 9;18(9):e0012462. doi: 10.1371/journal.pntd.0012462 (PMC11412511; doi:10.1371/journal.pntd.0012462)
Supplement: Attachment — Submitted filename: Author response to reviewers comments (a).docx [file pntd.0012462.s005.docx]

**Author response to reviewer’s comments**

|  | **Comment** | **Response** |
| --- | --- | --- |
| 1. | Reason for not including studies which included data sets from longitudinal studies following mass drug administration | - We excluded these articles because they did not provide the baseline data regarding the magnitude (prevalence) of the disease prior to the intervention.  - This information is very crucial since it provides the highlights on the actual state of the disease before intervention. However, all the publications containing baseline prevalence data were included. |
| 2. | Studies assessing genetic dynamics among *S. haematobium* populations | - The authors have now added *S. mansoni* in the mentioned criterion. However, during screen there was no publication assessing the genetic dynamics among *S. mansoni.*  - The authors did not include these articles because they did not provide prevalence data that are crucial for this study. |
| 3. | Can the authors explain why they chose to subgroup the publication years as they did | - The number of studies per year was low to include each year in the sub group analysis. However, by grouping them in five-year interval will provide enough studies to give a more realistic estimate of the prevalence. - In addition, grouping studies helped to provide an insight regarding the trend of the disease in five years interval (whether is increasing or decreasing). |
| 4. | Table 1 – Can this table be ordered by either author name (alphabetically) or year or sample size? This would make it easier to read. | - The table has been ordered alphabetically based on the authors names as recommended by reviewers. |
| 4. | Can the authors explain how they ensured that articles were not using the same datasets and add it to the exclusion criterion | - Articles duplicating part of or all dataset from the project or research group were excluded from the study.  - This criterion was also added in the exclusion criteria for this study as suggested by reviewer.  - Identification of the duplicated data was achieved by doing carefully comparison of the reported data from two or more articles from similar project or research group.  - Carefully examination of the study design was used to get a clue regarding articles containing dataset from similar project, study population, sample size and time of data collection. |
| 5. | Line 346 states that the high prevalence of *S. mansoni* prevalence can be attributed to the tendency of people to defecate near water bodies – where in this review did the authors find this? If this was from other studies, please be clear of this. | - The statement was cited as recommended by reviewers |
| 6. | Line 352 – the authors should be careful with what they say their results show – an increase of pooled prevalence from all parasite species over all regions does not necessarily show an inefficiency of PZQ by MDA as a disease control and elimination strategy. As this is pooled prevalence, it could be that some regions have responded very well to MDA, but this is diluted by other regions which either had not been involved in treatment strategies or had low coverage due to unknown reasons – but we cannot know this from the data presented here. Furthermore, some regions will have had additional control strategies such as snail control etc. but this is not accounted for in the results. The authors themselves state that the control interventions initiated by ZEST in the islands has reduced disease burden from above 50% to below 5%, therefore contradicting the aforementioned claim that control interventions are not working in the country | The sentence was corrected as recommended by reviewer. |
| 7. | High prevalence does not necessarily mean high morbidity, the authors say Tanzania marks the second country in terms of high disease morbidity rate, however neither reference cited here support this claim. Reference 6 states that there is significant morbidity in Tanzania, but they cite a different study to support this and do not report any morbidity results themselves. Reference 68 does not specifically talk about morbidity in Tanzania. | The sentence was corrected as suggested by the reviewer. |
| 8. | However, I think some of the claims made by the authors of what these data can actually show are not founded in the way the results are presented. | The statements have been carefully revised and rephrased as suggested by the reviewer. |
| 9. | The authors have addressed the comments raised in my previous review report, and I see significant improvement. However, before being accepted, I recommend that the manuscript be revised by a professional English language editor | The manuscript was proofread by a professional English language editor, as suggested by the reviewer. |
